# A Scoping Review of Nursing Leadership Role in Global Health: Challenges and Opportunities

**DOI:** 10.1111/nhs.70377

**Published:** 2026-07-08

**Authors:** Cassiane Santana Lemos, Mayara Spin, Raquel Rondina Pupo da Silveira, Vanessa de Brito Poveda, Ana Maria Müller de Magalhães, Maria Alice Dias da Silva Lima, Miguel Lucas Silva da Paixão, Silvia Cristina Garcia Carvalho, Nancy Reynolds

**Affiliations:** ^1^ Nursing Department The Medical School (FMB) of São Paulo State University (Unesp) Botucatu Sao Paulo Brazil; ^2^ School of Nursing‐ Sao Paulo University Sao Paulo Brazil; ^3^ School of Nursing‐ The Federal University of Rio Grande do Sul (UFRGS) Porto Alegre Rio Grande do Sul Brazil; ^4^ Johns Hopkins School of Nursing Baltimore Maryland USA

**Keywords:** Global Health, leadership, nurse's role

## Abstract

Nursing leadership plays a critical role in ensuring healthcare quality and safety while also contributing to improving the population health outcomes in the context of global health. The aim was to map the literature on the role of nursing leadership in global health. A scoping review was constructed using databases MEDLINE, EMBASE, Scopus, Web of Science, CINAHL, BDEnf, LILACS, and SciELO through September 2024. The review followed the PRISMA‐ScR guidelines, the JBI methodology, and the Population, Context and Concept (PCC) framework. The search identified 557 articles. After excluding duplicates and screening titles and abstracts, 41 full‐text articles were assessed for eligibility, resulting in a sample of 22 articles, which included nursing leadership in policy (14;63.6%), care (7;31.8%), and education (6;27.3%). The findings underscore the importance of nursing leadership in strengthening global health, but also reveal persistent challenges across care delivery, educational backgrounds, and political engagement. Therefore, nursing leadership in global health is supported by three interconnected pillars: policy, care, and education. The results highlight the need to advance nursing leadership capacity to deal with global health demands.

## Keypoints

- Key findings show that nursing leadership in global health is built on three core pillars: policy, clinical care, and education.
- Nursing leadership in global health holds great potential for contributing to worldwide healthcare management, but still faces challenges in gaining full recognition.
- There is a need to promote training and empowerment of nurse leaders so they can take on strategic political roles at both national and international levels.

## 1 Introduction

The process of globalization, characterized by the opening of trade borders, increased communication among nations, and advances in health technologies, has contributed to the development of global health as a multidisciplinary concept. This concept encompasses not only medical and biological aspects, but also cultural, environmental, socioeconomic, and political dimensions on a global scale. It is grounded in ethical principles such as cooperation, equal access to health care, and social justice (Fortes and Ribeiro 2014; Wilson et al. 2016).

Global health, rooted in public and international health principles, aims to promote community‐based actions focused on health promotion, disease prevention, care delivery, and recovery. It adopts a transnational perspective that transcends geographical borders, positioning itself as a core element in the formulation of public policies that emphasize equity, awareness, and international cooperation (Fortes and Ribeiro 2014; Wilson et al. 2016).

The active participation of healthcare professionals, policymakers, researchers, and civil society is necessary for ensuring the legitimacy and effectiveness of decisions made by organizations such as the World Health Organization (WHO), the Pan American Health Organization (PAHO), professional associations, and collegiate bodies. These stakeholders are critical in coordinating responses to health crises, formulating evidence‐based policies, advocating for health rights, and advancing sustainable development (Organização Pan‐Americana da Saúde 2022; World Health Organization 2025).

Within this context, nurses serve as advocates, managers, educators, researchers, and leaders. They play a vital role in health care delivery and in ensuring universal health coverage. Given these positions and their holistic, person‐centered approach to care, nurses are uniquely positioned to influence politics, drive educational reform, contribute to economic and environmental agendas, and address social determinants of health. Moreover, as the largest workforce within the healthcare system, nurses' potential to impact population health outcomes is substantial (Wilson et al. 2016; Global Strategic Directions for Nursing and Midwifery 2021–2025 2021).

Regardless of their area of expertise, whether clinical practice, education, administration, or policymaking, nurses possess the knowledge and experience necessary to serve as agents of change (World Health Organization 2020). Their contributions are pivotal in achieving universal health coverage, influencing policy development, resource allocation, and addressing a wide range of global health priorities, including mental health, non‐communicable diseases, emergency preparedness and response, patient safety, and integrated and people‐centered care, contributing to promoting population health and well‐being (Global Strategic Directions for Nursing and Midwifery 2021–2025 2021; Mendes et al. 2016a; Kickbusch and Liu 2022).

Nursing's role aligns directly with Sustainable Development Goal 3 (SDG 3) and intersects with several, depending on the area of practice. Nurses are instrumental in implementing integrated and people‐centered care models, facilitating access to health care and technologies, and promoting health (Wilson et al. 2016; Global Strategic Directions for Nursing and Midwifery 2021–2025 2021; Kickbusch and Liu 2022).

The interconnection of health, globalization, and foreign policy has led to the rise of health diplomacy, which mobilizes state and non‐state actors and intergovernmental organizations to coordinate policies, agreements, and interventions in global health (Falqui et al. 2024; Ruckert et al. 2016; Marc et al. 2019). This context involves a variety of stakeholders, including nursing professionals. Their contributions enhance the formulation and implementation of equitable health policies and align with the core principles of the SDGs (Hunter et al. 2013; Sharafi et al. 2021; Institute of Medicine (US) Committee on the Robert Wood Johnson Foundation Initiative on the Future of Nursing, at the Institute of Medicine 2011).

Despite these contributions, nursing leadership faces significant barriers including professional bias, lack of formal recognition, and limited opportunities for training in areas such as management, policy, and negotiation. These challenges hinder the development of essential leadership competencies, including critical thinking, problem‐solving, and strategic decision‐making, thereby restricting nurses' participation in key decision‐making forums (Institute of Medicine (US) Committee on the Robert Wood Johnson Foundation Initiative on the Future of Nursing, at the Institute of Medicine 2011; International Council of Nurses (Stewart and ICN 2022).

Given the importance of nursing leadership in ensuring healthcare quality and safety, it is essential to better understand and support nurses' roles in global health. Understanding this environment can provide a clearer picture of nursing practice, as well as the barriers and challenges that must be overcome to empower these professionals to influence health system policy decisions. Thus, this study aimed to map the existing scientific literature on nursing leadership roles in the context of global health.

## 2 Methodology

This scoping review was developed according to JBI (Peters et al. 2020) methodology and was registered on the Open Science Framework (https://doi.org/10.17605/OSF.IO/8ZYXB). The review process adhered to the Preferred Reporting Items for Systematic Reviews and Meta‐Analyses extension for Scoping Reviews (PRISMA‐ScR) guidelines (Tricco et al. 2018) (File S1).

### 2.1 Research Question

The guiding question of the review was: What is the ROLE OF nurses' leadership in the context of global health?

To define the research question and eligibility criteria, the Population‐Concept‐Context (PCC) framework was used (Peters et al. 2020): Population (P) = nurses; Concept (C) = nursing leadership; Context (C) = global health.

The review considered leadership at any level of nursing practice, which could include primary care, manager, or acute care.

### 2.2 Eligibility Criteria

We included original articles, theses, dissertations, reviews, argumentative essays, and monographs published in full until September 2024 that addressed the guiding question, aimed to map all concepts of nursing leadership, and considered that scoping reviews can include all kinds of available literature. No language restrictions were applied. Simple or expanded abstracts, posters, editorials, and book chapters were excluded.

### 2.3 Search Strategy

Descriptors were selected using the Virtual Health Library (VHL) Health Sciences Descriptors (DeCS), Medical Subject Headings (MeSH), and Emtree. Boolean operators AND and OR were used with the terms “Global Health”, “Leadership”, and “Nurse's Role” adapted for each database (File S2).

Databases searched included the Medical Literature Analysis and Retrieval System online (MEDLINE/PubMed), Excerpta Medica Database (EMBASE), Scopus, Web of Science (WoS), Cumulative Index to Nursing and Allied Health Literature (CINAHL), *Base de Dados em Enfermagem* (BDEnf), and Latin American and Caribbean Literature in Health Sciences (LILACS), through VHL, and the Scientific Electronic Library Online (SciELO), through the WoS interface. The searches were conducted from October 2023 to September 2024.

### 2.4 Data Selection and Extraction

Two independent reviewers using Rayyan software, developed by the Qatar Computing Research Institute, to screen titles and abstracts. Duplicates were removed and potentially eligible articles were assessed in full. A third reviewer resolved any discrepancies.

Data were extracted using an adapted JBI (Peters et al. 2020) instrument and included article ID (identification number), authorship, country, study design, context, participants, main topic, and results, as well as barriers and challenges in leadership roles.

According to the WHO's Global Strategic Directions for Nursing and Midwifery (2021–2025), policy priorities for the nursing profession should include educational initiatives, job availability, leadership skills development, and regulation of service delivery (Global Strategic Directions for Nursing and Midwifery 2021–2025 2021). The articles were classified into three thematic areas: education, policy, and care.

Nursing education is a multifaceted topic, covering the development of scientific and technical knowledge, as well as leadership skills to help manage nursing practice and enhance nurses' involvement in teamwork. In addition, policies in the nursing area include rules and laws that define nursing practice and competencies in healthcare settings, empower nurses to advocate for better working conditions, support healthcare system development, and improve patient quality of care (Global Strategic Directions for Nursing and Midwifery 2021–2025 2021; World Health Organization 2020; Mendes et al. 2016a).

**FIGURE 1:**
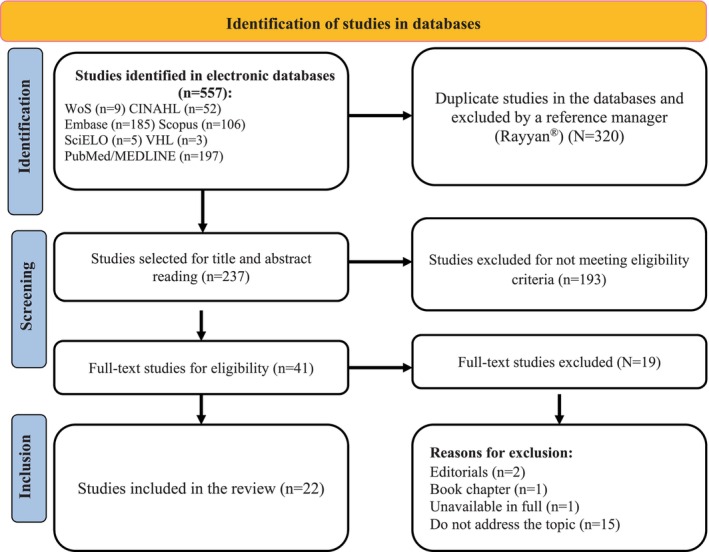
PRISMA‐ScR Flowchart (Tricco et al. 2018) adapted. Botucatu—SP, Brazil (2024).

The initial search yielded 557 articles: 197 from MEDLINE/PubMed, 185 from Embase, 106 from Scopus, 52 from CINAHL, nine from WoS, five from SciELO, and 3 from VHL. After removing 320 duplicates and excluding 193 irrelevant articles (File S3), 41 articles were reviewed in full. Of these, 22 met the inclusion criteria and were included in the final review (Figure 1).

### 2.5 Data Analysis and Presentation

The data were analyzed descriptively, and the results were presented through charts and tables.

## 3 Results

The 22 studies included were conducted in the United States (*n* = 11; 50%) (Opollo et al. 2012; Nicholas and Breakey 2017; Schenk 2019; Buckner et al. 2014; Schneider et al. 2009; Kim et al. 2006; Potter 2019; Duncan and Whyte 2010; Porta et al. 2019; Solheim et al. 2024; Zalon et al. 2024), Brazil (*n* = 3; 13.6%) (Salvage and White 2020; Mendes et al. 2016b; Mendes et al. 2020), Switzerland (*n* = 2; 9.1%) (Stewart and Halpin 2019; Salvage et al. 2019), and Australia (*n* = 2; 9.1%) (McMurray 2007; Dawson et al. 2015). The studies were published between 2006 and 2024, with the highest publication density in 2019 (*n* = 7; 31.8%) (Salvage and White 2019; Schenk 2019; Stewart and Halpin 2019; Salvage et al. 2019; Klopper et al. 2020; Potter 2019; Porta et al. 2019).

Table 1 presents study details, including author, database, country, study design, and main findings.

**TABLE 1:** Studies selected according to author/year of publication and origin, study design, context, participants, main topic, results, and challenges of leadership role. Botucatu, SP, Brazil (2024).

Author/year	Origin	Study design	Context	Participants	Main topic	Main results	Challenges for global nursing leadership role
Kim et al. (2006)	United States	Qualitative study	Nurse leader participants from international organizations	17 nurses	Policy	Developing global nursing leaders is an ongoing process that requires the development of cognitive and cultural skills. Vision and interpersonal skills are critical to shaping the future of global nursing leadership. Personal attributes, family upbringing, professional experiences, and key leadership positions in international organizations were important influences on global leadership development.	Formal and informal education for global competencies Cultural and cognitive knowledge to become a leadership International experiences and partnership
McMurray (2007)	Australia	Argumentative essay	Primary care	No applicable	Care	Nurses and their leadership skills are important for combating inequality and promoting equity in primary care, seeking solutions to complex challenges, and strengthening partnerships.	Evaluation of context and communities needs To develop actions directed to inequalities conditions and limited resources
Schneider et al. (2009)	United States	Exploratory descriptive	Urban sustainability and human health	No applicable	Policy	Nursing influences environmental health, defending health rights protection through professionals' education, motivation, and encouragement in sustainable best practices.	Nursing professionals as protagonists of human health and sustainability in urban areas
Duncan and Whyte 2010)	United States	Argumentative essay	Primary care and Academic context	No applicable	Policy	Nursing leadership is essential to transforming health systems. Nurses' active participation at all levels of management, from policy development to research, ensures that the community's voice is heard and that healthcare services are aligned with the population's real needs.	Nursing actions to promote universal health coverage, including leadership in political scenarios and human resources.
Opollo et al. (2012)	United States	Exploratory descriptive	Academic context	No applicable	Education Policy	Nurses as leaders in promoting global health in research, education, policy, and care, with a focus on international partnerships and cultural exchange.	International communication and partnership Development of global health research and permanent education
Buckner et al. (2014)	United States	Exploratory descriptive	Members of the leadership of Sigma Theta Tau International, Regional Chapter Coordinating Committee	Six nurses	Care Policy	Nursing leadership, through creativity, change, collaboration, community, context, and courage, drives global health by strengthening networks and fostering the development of new leaders.	Leadership development faces: workplace pressures, time constraints, political issues
Dawson et al. (2015)	Australia	Systematic review	Primary care	Cinahl, Medline, Pubmed, Scopus, Current Contents Connect, Web of Science, Proquest Health & Medicine, Science Direct and Google Scholar portals/databases	Education Care	Nurses as leaders in planning actions to prevent health inequality and universal access to vulnerable populations.	Multitasking health professional and difficulties in retaining nurses and midwives who work with vulnerable populations.
Mendes et al. (2016b)	Brazil	Qualitative documentary	Universal Health Coverage	No applicable	Care	Nursing, a fundamental pillar for universal health coverage, works on three fronts: education, partnerships, and leadership. Training qualified professionals and building political dialogues and innovation in management are essential to guarantee equitable and comprehensive access to health.	Protection against financial risks, and to design indicators and data to monitor the progress of the universal coverage policy through nursing actions.
Nicholas and Breakey (2017)	United States	Narrative review	Climate change and environmental health	No applicable	Policy	Climate change and nursing leadership role in environmental health education, research on climate impacts on health, and action in public policies for sustainability and climate justice.	Recognition of climate justice, climate change, and climate stewardship for environmental and community health
Salvage and White (2019)	United Kingdom	Argumentative essay	Political settings	No applicable	Policy	Nursing as a contributor to the development of evidence‐based public policies for health promotion, gender equality, poverty reduction, and environmental sustainability.	Active participation of nurses in policy‐making and decision‐making
Schenk (2019)	United States	Narrative review	Planetary health	No applicable	Policy	Nurses in the development of sustainability action plans, through their leadership skills and advocacy for environmental management, strategic planning, and community leadership, discussing healthcare and environmental pollution.	Nursing actions that contribute to reducing environmental pollution
Stewart and Halpin (2019)	Switzerland	Argumentative essay	Political settings	No applicable	Care	Nurses as leaders in crucial areas of global health, such as sanitation, mental health, human rights, and the environment. This leadership is essential to building stronger and more resilient health systems, promoting significant and lasting changes.	Active participation of nurses in policy‐making and decision‐making
Salvage et al. (2019)	Switzerland	Exploratory descriptive	Leadership Program	No applicable	Policy	The occupation of political positions by nurses is essential to ensure the implementation of public policies that promote population health, improve the quality of care, and value the nursing profession.	Structured training programs for nursing leadership
Klopper et al. (2020)	South Africa	Narrative review	Sigma Theta Tau International core panel‐ Global Advisory Panel on the Future of Nursing and Midwifery (GAPFON)	No applicable	Care Education Policy	Nurses as leaders in building fairer and more equitable health systems. Through transformative education, participation in political decision‐making spaces, and the implementation of evidence‐based public policies, nurses can promote universal health coverage and improve the quality of care.	Nursing professional needs to improve policy, workforce, practice, education and research to further the global health issues
Potter (2019)	United States	Argumentative essay	Planetary health	No applicable	Education	Nursing, with its ability to care, innovate, and build relationships of trust, can be an agent of transformation in planetary crisis. By promoting health in an integrated manner and addressing social and environmental issues, nurses contribute to building a more just and sustainable world.	Nursing education to lead the transformation of planetary systems and health systems.
Porta et al. (2019)	United States	Narrative review	Sustainable Development Goals (SDGs)	No applicable	Education	By developing interprofessional and collaborative work strategies, nurse leaders should encourage research on the SDGs, shaping professionals prepared to face global challenges and promote health in an integrated manner.	Nursing strategies in education, workforce, research to achieve the SDGs
Mendes et al. (2020)	Brazil	Documentary descriptive	Sustainable Development Goals (SDGs)	No applicable	Care	Nursing leadership and active participation in the development and implementation of health policies are essential to transform the global health landscape. Investment is needed in developing global programs that strengthen nursing leadership at all levels, ensuring that nursing's voice is heard and that health goals are achieved.	Investment in health and nursing workforces to achieve the SDGs
Salvage and White (2020)	Brazil	Exploratory descriptive	Sustainable Development Goals (SDGs)	No applicable	Policy	Nurses as leaders in health promotion, acting as a bridge between health systems and communities. By strengthening partnerships and basing their actions on scientific evidence, nurses can influence the formulation of public policies, reduce inequalities, and contribute to the achievement of the SDGs.	Active participation of nurses in policy‐making and decision‐making
Bryant‐Lukosius (2022)	Canada	Argumentative essay	Health systems	No applicable	Policy	Nurses as clinical leaders to strengthen health systems, promote best practice, continuing education and interprofessionality. Nurses contribute to the continuous improvement of care, advocacy of public policies and ensuring access to healthcare services.	Active participation of nurses in policy and decision‐making to advanced practice nursing leadership
Swapna et al. (2023)	India	Exploratory sequential mixed method	Hospitals, colleges and schools of nursing	60 Nurse administrators	Policy	To ensure nursing success in global health, it is crucial to invest in continuing education, workplace safety and adequate conditions, strengthening nurses' capacity to lead research, implement programs and influence policies.	Sustainable investments must be made in nursing workforce and infrastructure
Solheim et al. (2024)	United States	Exploratory descriptive	Global health (GH) and Planetary health (PH) interconnections	No applicable	Education	Strategies to academic nursing programs that can be applied to integrating GH and PH into their programs, including partnership, policy and research	Development of academic nursing programmes to lead, educate, discover and advocate for populations and planetary health.
Zalon et al. (2024)	United States	Narrative review	Political settings	No applicable	Policy	Nursing has the potential to drive policy changes that benefit patients and communities. In this context, a framework is proposed to nurses defines three essentials dimensions for policy: engagement (recognized influential, advocacy and literacy); partnership (broad‐based coalitions, interdisciplinary and single unit or community group), and reach (national/global, regional/state, local/unit/organization).	Active participation of nurses in policy and decision‐making

**FIGURE 2:**
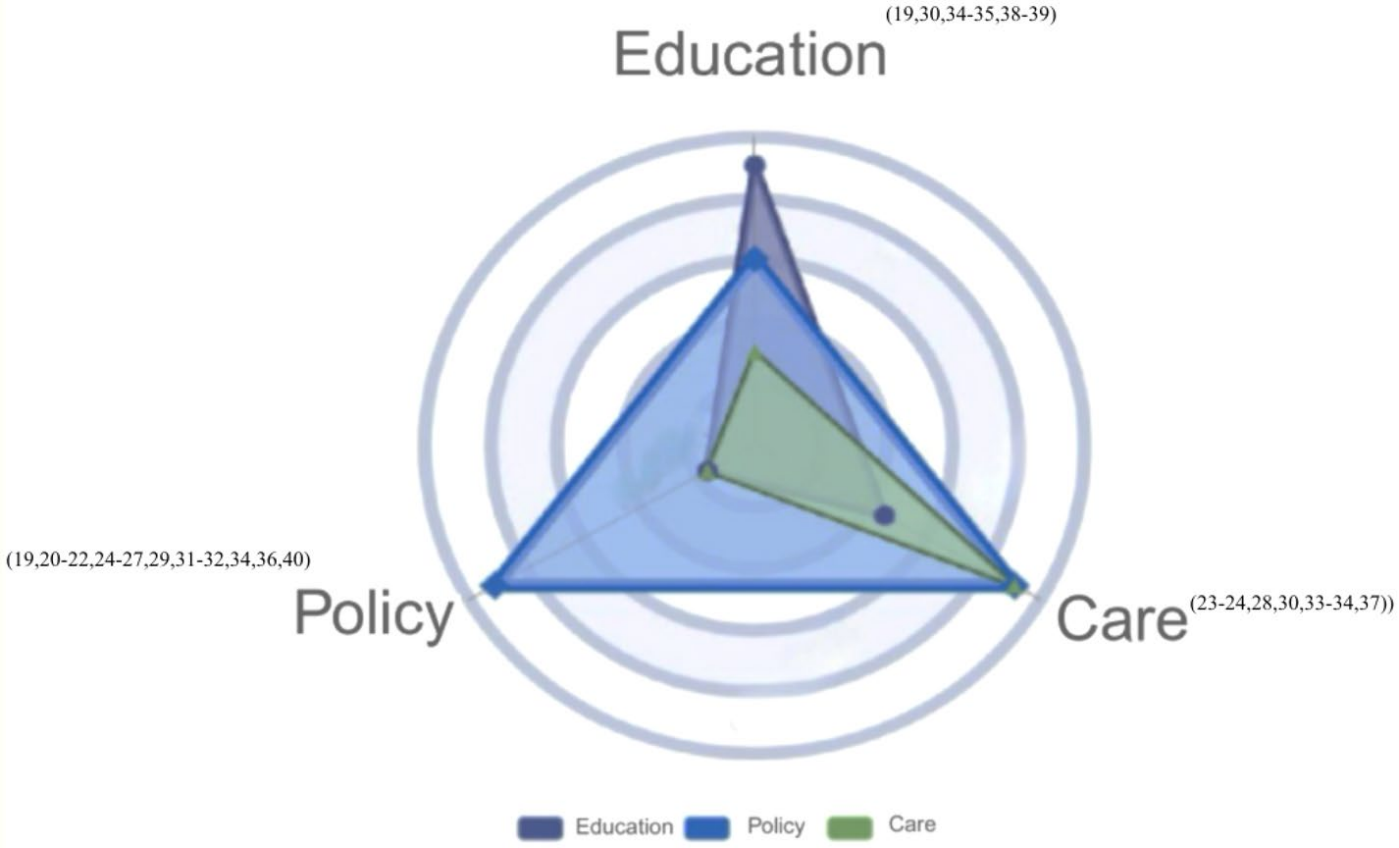
Graphical representation of nursing leadership role in education, policy, and care among the studies analyzed. Botucatu, SP, Brazil (2024).

Table 1 reveals that a considerable number of studies on leadership and global health are linked to policy‐making and decision‐making (Salvage and White 2019; Salvage and White 2020; Stewart and Halpin 2019; Bryant‐Lukosius 2022; Zalon et al. 2024), what can be observed is that 14 (63.6%) studies discussed policy; seven (31.8%) presented analyses of leadership in care; and six (27.3%) presented the role of nursing in education (Figure 2). Some studies showed more than one topic.

Educational leadership focused on (Opollo et al. 2012; Klopper et al. 2020; Solheim et al. 2024) nurses' roles in teaching, mentoring, promoting continuing education, and preparing new professionals to engage with emerging scientific evidence, to improve care and global health outcomes through evidence‐based interventions (Opollo et al. 2012; Potter 2019). Another key focus was identifying health needs, especially among vulnerable populations, to inform responses to public health emergencies, such as disasters, thereby improving health outcomes (Dawson et al. 2015; Porta et al. 2019).

Some Studies (Opollo et al. 2012; Salvage and White 2019; Salvage and White 2020) Addressed the Close Relationship Between Education and Policy Roles, Highlighting the Importance of Encouraging and Training Nurses and Students to Participate in Leadership and Decision‐Making, as Well as Occupying Political Roles, Which Can Contribute to Greater Adherence of This Professional Class to Decision‐Making Positions.

Studies Consistently Emphasize That Continuing Education for Nurses and the Integration of Policy and Educational Competencies Are Essential for Improving Healthcare Services (Dawson et al. 2015; Mendes et al. 2016b; Klopper et al. 2020; Potter 2019). They Also Underscore the Importance of Transformative and Interprofessional Education in Strengthening Health Systems, Informing the Development of Evidence‐Based Policies and Improving Population Health and Quality of Life.

Nursing leadership in health policy, global health advocacy, and strategic action planning emerged as recurring themes (Mendes et al. 2016b; Klopper et al. 2020; Potter 2019), affirming the importance of nurses as active contributors to health policy formation, advocacy, and global health promotion (Opollo et al. 2012; Nicholas and Breakey 2017; Salvage and White 2019; Salvage and White 2020; Mendes et al. 2016b). Across these studies, authors emphasize the need to empower nurses to lead political change, engage in public policy processes, and serve as leaders in global health, promoting equity, environmental health, and social justice (Salvage and White 2019; Salvage and White 2020; Stewart and Halpin 2019; Bryant‐Lukosius 2022; Zalon et al. 2024).

Another recurring theme is preparing nurses for leadership positions within international organizations and global policy platforms. Three studies (13.6%) (Buckner et al. 2014; Kim et al. 2006; Porta et al. 2019) address opportunities for nurses to strategically position themselves in these settings by cultivating global competencies and building partnerships that enhance their influence on global health leadership. These studies stress to the authors the importance of both professional experience and sustained training in equipping nurses for such roles.

Political empowerment of nurses is another key area identified in three studies (13.6%) (Salvage and White 2020; Rajaguru et al. 2022), which emphasize that greater political engagement among nurses is essential for advancing and defending effective public health policies.

Among the identified leadership contributions, health system strengthening stands out as a major area of impact. Three studies (13.6%) (Stewart and Halpin 2019; Mendes et al. 2016b; Klopper et al. 2020) describe how nurses contribute by expanding community access to care and implementing logistical and structural reforms in service delivery. Additionally, nurses were recognized as leading actors in efforts to address social inequalities and promote equity and inclusion in healthcare, as noted in two studies (9%) (McMurray 2007; Klopper et al. 2020). Leadership in the workplace was also associated with enhanced social inclusion, greater social justice, and the promotion of human rights, as noted in two studies (Stewart and Halpin 2019; Porta et al. 2019).

Educational leadership also featured prominently in the reviewed studies. Two studies highlighted how nurses promote evidence‐based practice by implementing innovative care models and professional standards in their practice environments (McMurray 2007; Bryant‐Lukosius 2022). Similarly, other studies (Mendes et al. 2016b; Klopper et al. 2020) identified interprofessional team empowerment and nurse‐led continuing education as effective strategies to foster integration and improve health outcomes. Lastly, communication, collaboration, and the establishment of interprofessional partnerships were recognized as key mechanisms for driving improvements in care quality (Buckner et al. 2014; Porta et al. 2019).

## 4 Discussion

This review showed that, for nurses to effectively serve as leaders and organizational partners, certain areas need improvement, including education, policy, and care practices. Such transformation should prioritize professional development, foster interprofessional collaboration, and promote inclusive decision‐making structures. Importantly, it requires valuing and enabling nurses' full participation in the development and implementation of equitable health policies, encouraging interprofessional collaboration, and ensuring their contributions are recognized and leveraged to shape fairer and more responsive health systems (Institute of Medicine (US) Committee on the Robert Wood Johnson Foundation Initiative on the Future of Nursing, at the Institute of Medicine 2011; World Health Organization 2014).

To advance nursing leadership, it must be prioritized and supported by local governments through social, cultural, and financial investments. However, political and organizational priorities may vary significantly between developed and developing countries. Countries such as South Korea (Lee et al. 2023), the United States (Solheim et al. 2024; Lynn et al. 2021), and England (Schleiff et al. 2021) have already made important progress by incorporating global health leadership content into nursing curricula.

This trend aligns with findings from the reviewed studies, which advocate active involvement of nursing professionals in developing and implementing practices that can be replicated locally and internationally (Opollo et al. 2012). These studies emphasize the need to educate nurses about their critical role in shaping public policies that reduce health inequalities and expand access to care (Dawson et al. 2015). Accordingly, higher education programs must prepare nurses not only to engage in political decision‐making and drive transformative changes that foster more equitable health systems (Klopper et al. 2020), but also to understand diversity and culture, which influence healthcare and local, regional, and global government policies.

Notably, most countries introduce global health and leadership topics at the graduate level, with limited integration into foundational nursing education (Zago et al. 2023; Hwang and Jo 2020). This omission represents a missed opportunity to equip all nurses—not just those pursuing advanced degrees—with competencies needed for global engagement. In underdeveloped or developing countries, contextual challenges such as health system limitations, resource constraints, and competing priorities often result in curricula that are narrowly focused on immediate regional needs, limiting broader training in global competencies and perspectives (Holst 2020; Rajaguru et al. 2022).

While organizations such as the WHO (World Health Organization 2020) recognize the need for well‐trained and motivated professionals to deliver evidence‐based care that responds to global health challenges, existing studies reveal a significant gap in the education of nurses prepared to act critically, reflectively, and collaboratively, and to work within globally integrated frameworks (Holst 2020; Kang et al. 2021; Lee and Quinn 2021). This disconnect underscores the urgency of rethinking nursing education to align with the demands of a rapidly evolving global health landscape.

In contrast, international organizations such as PAHO and the Global Nursing Leadership Institute have played a transformative role in advancing nurses' political and social representation worldwide. Through initiatives such as the Nursing Now campaign, these organizations have helped elevate nurses' visibility and influence in leadership and policy arenas. Hence, as emphasized by several authors (Duncan and Whyte 2010), nurses, given their frontline role in healthcare delivery, must also have a seat at the table where decisions are made, enabling them to voice the challenges they encounter in practice and contribute meaningfully to policy development.

Improving clinical practice through the synthesis and implementation of evidence, with trained nurses as clinical leaders for change, may yield results limited to points of care but also create the necessary environment for the development of clinical leadership skills, visibility, and influence in other healthcare delivery settings. In this sense, international organizations such as the JBI have contributed by offering tools that enable healthcare professionals, including those for clinical leadership development (Lockwood and Ivers 2023). This can contribute to the prominence of nurses in healthcare settings, as well as the recognition of teamwork and societal roles.

Leadership is intrinsic to the nursing profession and is essential to addressing social barriers and health challenges worldwide. By promoting social justice and bridging gaps in access and comprehensiveness, nurses play a pivotal role in advancing equitable healthcare systems (Buckner et al. 2014; Schneider et al. 2009; Stewart and Halpin 2019). However, they have faced significant challenges, including a lack of recognition for their work by society (Netto 2024), a shortage of staff (Royal College of Nursing, Nursing Workforce Academy, n.d.), and inadequate wages (OECD 2025). These issues have led many professionals to leave the profession or migrate to other countries in search of better working conditions and remuneration (World Health Organization (WHO) 2025).

Despite persistent challenges, nurses' engagement in political and policy processes has proven to be valuable as a bridge between health systems and the communities they serve. Grounded in a humanitarian ethos, nurses are educated to respond to both individual and community needs, addressing critical issues such as sanitation, mental health, human rights, social inequalities, and care of vulnerable populations. This positions them uniquely to foster social well‐being, improve healthcare access, and advocate for equity in health services (Salvage and White 2020; Stewart and Halpin 2019; Klopper et al. 2020).

The challenges of nursing leadership often originate in the day‐to‐day realities of healthcare delivery and micropolicy contexts, as presented in different studies in this review (Salvage and White 2019; Salvage and White 2020; Stewart and Halpin 2019; Bryant‐Lukosius 2022; Zalon et al. 2024). In this sense, nurses must assert leadership within their practice environments, serving as informed advocates who draw on scientific evidence and socially responsive care models. By doing so, they can drive meaningful improvements in healthcare delivery and outcomes within their specific contexts (Salvage and White 2020; Stewart and Halpin 2019; Bryant‐Lukosius 2022).

Significantly, continuing professional development in practice guides nurses' practice in line with the best evidence. This contributes to leadership in care management and decision‐making, thereby reinforcing the role of nurses in multidisciplinary teams and health systems (Mlambo et al. 2021).

In this context, amplifying the nursing voice begins with advocating for the social rights of both patients and colleagues within local communities. Nurses must be equipped to assume active roles in transforming health institutions and service delivery. Achieving this requires significant progress in creating supportive professional practice environments, fostering collaborative approaches, advancing interprofessional education and management, and, crucially, addressing the persistent lack of institutional support for nursing leadership development (McMurray 2007; Buckner et al. 2014; Klopper et al. 2020).

The integration of policy, education, and care is fundamental to strengthening nurses' voices across diverse countries, contexts, and health systems. Building a robust foundation for nursing leadership demands strategic collaboration among international and national organizations, and academic and research institutions. Such partnerships are critical to cultivating a globally competent nursing workforce, one that is not only knowledgeable and technically proficient, but also empowered to fulfill its essential role in reinforcing and transforming health systems (Dupin et al. 2020).

## 5 Review Limitations

Most of the articles were written by authors living in North America and Europe. Therefore, there is a limitation to the generalization of results to underdeveloped and/or developing countries, as the reality of nursing practice in these regions may differ with respect to laws and regulations, which can influence nurses' leadership and alignment with the global health agenda.

However, it is acknowledged that this limitation reflects a broader pattern observed across multiple fields of knowledge in health and nursing, in which scholarly publications are predominantly concentrated in high‐income countries, while comparatively less attention has been directed toward low‐ and middle‐income countries (LMICs).

Future studies, especially in LMICs, are important for evaluating the regional reality and proposing improvements to nursing roles from these local perspectives. These initiatives are fundamental because professional competencies and laws vary from country to country, making it impossible to generalize about roles and possibilities of practice.

## 6 Final Considerations

This review showed that current scientific production on nursing leadership roles in the context of global health directs action toward political, healthcare, and educational contexts, highlighting their inseparability and the topic's challenges. Given this scenario, further research is needed to evaluate the effectiveness of initiatives aimed at improving the role of nurses' leadership globally, including in low‐ and middle‐income countries.

Thus, global health priorities in the nursing area can be proposed from a broadly inclusive perspective, grounded in local realities and with aims achievable by all countries while respecting regional legislation, population health needs, and financial resources.

## 7 Relevance for Clinical Practice

Empowering nurses with global health concepts is the cornerstone of improving leadership in practice and changing professionals' reality. In this sense, nurses can be better supported to assume decision‐making roles and contribute significantly to health outcomes.

## Author Contributions

**Cassiane Santana Lemos:** conceptualization, investigation, writing – original draft, methodology, validation, visualization, writing – review and editing, project administration, formal analysis, supervision, resources. **Miguel Lucas Silva da Paixão:** investigation, writing – original draft, methodology, validation, visualization, writing – review and editing, formal analysis, data curation. **Mayara Spin:** investigation, writing – original draft, methodology, validation, visualization, writing – review and editing, formal analysis, data curation. **Ana Maria Müller de Magalhães:** writing – original draft, methodology, validation, visualization, writing – review and editing, formal analysis, project administration, data curation, supervision, resources. **Raquel Rondina Pupo da Silveira:** investigation, writing – original draft, methodology, validation, visualization, writing – review and editing, formal analysis, data curation. **Silvia Cristina Garcia Carvalho:** investigation, writing – original draft, methodology, validation, visualization, writing – review and editing, formal analysis, data curation. **Nancy Reynolds:** writing – original draft, writing – review and editing, supervision, data curation. **Maria Alice Dias da Silva Lima:** writing – original draft, writing – review and editing, supervision, data curation. **Vanessa de Brito Poveda:** conceptualization, writing – original draft, methodology, validation, visualization, writing – review and editing, formal analysis, project administration, data curation, supervision, resources.

## Funding

The authors have nothing to report.

## Ethics Statement

The authors have nothing to report.

## Conflicts of Interest

The authors declare no conflicts of interest.

## Supporting information


**File S1:** Prisma‐ScR Checklist.

**File S2:** Search strategy.

**File S3:** Reasons for exclusion.

## Data Availability

Data sharing not applicable to this article as no datasets were generated or analysed during the current study.

## References

[nhs70377-bib-0001] Bryant‐Lukosius, D. 2022. “Future Leadership.” Investigación en Enfermería: Imagen y Desarrollo 24, no. 1: 1–2. 10.11144/Javeriana.ie24.flap.

[nhs70377-bib-0002] Buckner, E. B. , D. J. Anderson , N. Garzon , T. B. Hafsteinsdóttir , C. K. Lai , and R. Roshan . 2014. “Perspectives on Global Nursing Leadership: International Experiences From the Field.” International Nursing Review 61, no. 4: 463–471. 10.1111/inr.12139. 25411072

[nhs70377-bib-0003] Dawson, A. J. , A. M. Nkowane , and A. Whelan . 2015. “Approaches to Improving the Contribution of the Nursing and Midwifery Workforce to Increasing Universal Access to Primary Health Care for Vulnerable Populations: A Systematic Review.” Human Resources for Health 13: 97. 10.1186/s12960-015-0096-1. 26684471 PMC4683743

[nhs70377-bib-0004] Duncan, S. , and N. Whyte . 2010. “Global Leadership Priorities for Canadian Nursing: A Perspective on the ICN 24th Quadrennial Congress, Durban, South Africa.” Nursing Leadership (Toronto, Ont.) 23, no. 1: 16–21. 10.12927/cjnl.2010.21725. 20383076

[nhs70377-bib-0005] Dupin, C. M. , M. Pinon , K. Jaggi , C. Teixera , A. Sagne , and N. Delicado . 2020. “Public Health Nursing Education Viewed Through the Lens of Superdiversity: A Resource for Global Health.” BMC Nursing 19: 18. 10.1186/s12912-020-00411-3. 32206035 PMC7083057

[nhs70377-bib-0006] Falqui, L. , F. Li , and Y. Xue . 2024. “Global Health Diplomacy in Humanitarian Action.” Conflict and Health 18, no. 1: 46. 10.1186/s13031-024-00605-5. 39026338 PMC11264823

[nhs70377-bib-0007] Fortes, P. A. d. C. , and H. Ribeiro . 2014. “Saúde Global em tempos de globalização.” Saúde e Sociedade 23, no. 2: 366–375.

[nhs70377-bib-0008] Global Strategic Directions for Nursing and Midwifery 2021–2025. 2021. World Health Organization License: CC BY‐NC‐SA 3.0 IGO, https://iris.who.int/server/api/core/bitstreams/11723bae‐3fd4‐46f0‐a4ea‐9dc8fd153667/content.

[nhs70377-bib-0009] Holst, J. 2020. “Global Health – Emergence, Hegemonic Trends and Biomedical Reductionism.” Globalization and Health 16, no. 1: 42. 10.1186/s12992-020-00573-4. 32375801 PMC7201392

[nhs70377-bib-0010] Hunter, A. , L. Wilson , M. Stanhope , et al. 2013. “Global Health Diplomacy: An Integrative Review of the Literature and Implications for Nursing.” Nursing Outlook 61, no. 2: 85–92. 10.1016/j.outlook.2012.07.013. 22999856 PMC7118513

[nhs70377-bib-0011] Hwang, W. J. , and H. H. Jo . 2020. “Development and Application of a Program for Reinforcing Global Health Competencies in University Nursing Students.” Frontiers in Public Health 8: 263. 10.3389/fpubh.2020.00263. 32695741 PMC7338674

[nhs70377-bib-0012] Institute of Medicine (US) Committee on the Robert Wood Johnson Foundation Initiative on the Future of Nursing, at the Institute of Medicine . 2011. The Future of Nursing: Leading Change, Advancing Health. National Academies Press (US). 24983041

[nhs70377-bib-0014] Kang, J. , J. Song , and W. Noh . 2021. “Impact of Nurses' Compassion Competence and Transcultural Self‐Efficacy on Their Global Health Nursing Competency.” Applied Nursing Research 60: 151453. 10.1016/j.apnr.2021.151453. 34247789

[nhs70377-bib-0015] Kickbusch, I. , and A. Liu . 2022. “Global Health Diplomacy‐Reconstructing Power and Governance.” Lancet 399, no. 10341: 2156–2166. 10.1016/S0140-6736(22)00583-9. 35594877 PMC9113726

[nhs70377-bib-0016] Kim, M. J. , W. Woith , K. Otten , and B. J. McElmurry . 2006. “Global Nurse Leaders: Lessons From the Sages.” Advances in Nursing Science 29, no. 1: 27–42. 10.1097/00012272-200601000-00004. 16495686

[nhs70377-bib-0017] Klopper, H. C. , E. Madigan , C. Vlasich , et al. 2020. “Advancement of Global Health: Recommendations From the Global Advisory Panel on the Future of Nursing & Midwifery (GAPFON).” Journal of Advanced Nursing 76, no. 2: 741–748. 10.1111/jan.14254. 31657041

[nhs70377-bib-0018] Lee, A. , and M. Quinn . 2021. “Global Health Education in U.K. Universities.” Global Health Journal 5, no. 3: 155–162. 10.1016/j.glohj.2021.06.001. 34692173 PMC8523299

[nhs70377-bib-0019] Lee, S. , E. Kim , and J. Y. Yoon . 2023. “Global Health Competencies and Educational Needs for Nursing Students in South Korea.” Journal of Nursing Education 62, no. 2: 75–82. 10.3928/01484834-20221213-02. 36779893

[nhs70377-bib-0020] Lockwood, C. S. , and N. M. Ivers . 2023. “Implementation Science: A Primer.” JBI Evidence Implementation 21, no. 4: 307–309. 10.1097/XEB.0000000000000398. 38031899

[nhs70377-bib-0021] Lynn, K. A. , T. McKinnon , E. Madigan , and J. J. Fitzpatrick . 2021. “Assessment of Global Health Competence of Nursing Faculty in Prelicensure Programs.” Journal of Nursing Education 60, no. 1: 20–24. 10.3928/01484834-20201217-05. 33400803

[nhs70377-bib-0022] Marc, M. , A. Bartosiewicz , J. Burzynska , Z. Chmiel , and P. Januszewicz . 2019. “A Nursing Shortage ‐ a Prospect of Global and Local Policies.” International Nursing Review 66, no. 1: 9–16. 10.1111/inr.12473. 30039849

[nhs70377-bib-0023] McMurray, A. 2007. “Leadership in Primary Health Care: An International Perspective.” Contemporary Nurse 26, no. 1: 30–36. 10.5172/conu.2007.26.1.30. 18041981

[nhs70377-bib-0024] Mendes, I. A. C. , C. A. A. Ventura , M. C. N. Silva , V. L. Lunardi , I. R. Silva , and S. S. Santos . 2020. “Nursing Now and Always: Evidence for the Implementation of the Nursing Now Campaign.” Revista Latino‐Americana de Enfermagem 28: e3388. 10.1590/1518-8345.4553.3388. 33174994 PMC7647412

[nhs70377-bib-0025] Mendes, I. A. C. , C. A. A. Ventura , M. A. Trevizan , L. M. Marchi‐Alves , and V. D. Souza‐Junior . 2016a. “Education, Leadership and Partnerships: Nursing Potential for Universal Health Coverage.” Revista Latino‐Americana de Enfermagem 24: e2673. 26959333 10.1590/1518-8345.1092.2673 PMC4822690

[nhs70377-bib-0026] Mendes, I. A. C. , C. A. A. Ventura , M. A. Trevizan , L. M. Marchi‐Alves , and V. D. Souza‐Junior . 2016b. “Educação, liderança e parcerias: potencialidades da enfermagem para a cobertura universal de saúde.” Revista Latino‐Americana de Enfermagem 24: e2673. 10.1590/1518-8345.1092.2673. 26959333 PMC4822690

[nhs70377-bib-0027] Mlambo, M. , C. Silén , and C. Mcgrath . 2021. “Lifelong Learning and Nurses'continuing Professional Development, a Metasynthesis of the Literature.” BMC Nursing 20: 62. 10.1186/s12912-021-00579-2. 33853599 PMC8045269

[nhs70377-bib-0028] Netto, J. J. M. 2024. “Invisibility and Devaluation of Nursing Work: Related Factors and Coping Strategies.” Investigación y Educación en Enfermería 42, no. 1: e01. 10.17533/udea.iee.v42n1e01 PMC11290902 39083813

[nhs70377-bib-0029] Nicholas, P. K. , and S. Breakey . 2017. “Climate Change, Climate Justice, and Environmental Health: Implications for the Nursing Profession.” Journal of Nursing Scholarship 49, no. 6: 606–616. 10.1111/jnu.12326. 28749596

[nhs70377-bib-0030] OECD . 2025. “Health at a Glance 2025: OECD Indicators.” OECD Publishing, Paris: 244. 10.1787/8f9e3f98-en.

[nhs70377-bib-0031] Opollo, J. G. , M. L. Bond , J. Gray , and V. J. Lail‐Davis . 2012. “Meeting Tomorrow's Health Care Needs Through Local and Global Involvement.” Journal of Continuing Education in Nursing 43, no. 2: 75–80. 10.3928/00220124-20111101-05. 22074213

[nhs70377-bib-0032] Organização Pan‐Americana da Saúde . 2022. Estratégia de Cooperação do País 2022–2027 ‐ Brasil. Versão revisada, OPAS, https://iris.paho.org/handle/10665.2/56315.

[nhs70377-bib-0033] Peters, M. D. J. , C. Godfrey , P. McInerney , Z. Munn , A. C. Tricco , and H. Khalil . 2020. “Scoping Reviews.” In JBI Manual for Evidence Synthesis, edited by E. Aromataris , C. Lockwood , K. Porritt , B. Pilla , and Z. Jordan . JBI, https://synthesismanual.jbi.global.

[nhs70377-bib-0034] Porta, C. M. , J. Disch , and N. Grumdahl . 2019. “Nursing Disruption for Achieving Sustainable Development Goals by 2030.” Nursing Administration Quarterly 43, no. 4: E1–E11. 10.1097/NAQ.0000000000000363. 31479063

[nhs70377-bib-0035] Potter, T. 2019. “Planetary Health: The Next Frontier in Nursing Education.” Creative Nursing 25, no. 3: 201–207. 10.1891/1078-4535.25.3.201. 31427415

[nhs70377-bib-0036] Rajaguru, V. , J. Oh , and M. Im . 2022. “Development and Evaluation of the Course on Global Health Nursing for Indian Nursing Students.” International Journal of Environmental Research and Public Health 19, no. 4: 1978. 10.3390/ijerph19041978. 35206165 PMC8871778

[nhs70377-bib-0037] Royal College of Nursing, Nursing Workforce Academy . n.d. “Safe staffing. Evaluating the Evidence for Mandatory Nurse‐to‐Patient Ratios.” file:///Users/cassi/Library/Mobile%20Documents/com~apple~CloudDocs/012–306.pdf.

[nhs70377-bib-0038] Ruckert, A. , R. Labonté , R. Lencucha , V. Runnels , and M. Gagnon . 2016. “Global Health Diplomacy: A Critical Review of the Literature.” Social Science & Medicine 155: 61–72. 10.1016/j.socscimed.2016.03.004. 26994358

[nhs70377-bib-0039] Salvage, J. , J. Montayre , and M. Gunn . 2019. “Being Effective at the Top Table: Developing Nurses' Policy Leadership Competencies.” International Nursing Review 66, no. 4: 449–452. 10.1111/inr.12567. 31721200

[nhs70377-bib-0040] Salvage, J. , and J. White . 2019. “Nursing Leadership and Health Policy: Everybody's Business.” International Nursing Review 66, no. 2: 147–150. 10.1111/inr.12523. 31124127

[nhs70377-bib-0041] Salvage, J. , and J. White . 2020. “Our Future Is Global: Nursing Leadership and Global Health.” Revista Latino‐Americana de Enfermagem 28: e3339. 10.1590/1518-8345.4542.3339. 32876292 PMC7458571

[nhs70377-bib-0042] Schenk, E. C. 2019. “Environmental Stewardship in Nursing: Introducing the ‘WE ACT‐*PLEASE*’ Framework.” Creative Nursing 25, no. 3: 222–231. 10.1891/1078-4535.25.3.222. 31427418

[nhs70377-bib-0043] Schleiff, M. J. , P. M. Mburugu , J. Cape , et al. 2021. “Training Curriculum, Skills, and Competencies for Global Health Leaders: Good Practices and Lessons Learned.” Annals of Global Health 87, no. 1: 64. 10.5334/aogh.3212. 34307067 PMC8284497

[nhs70377-bib-0044] Schneider, B. , N. Menzel , M. Clark , N. York , L. Candela , and Y. Xu . 2009. “Nursing's Leadership in Positioning Human Health at the Core of Urban Sustainability.” Nursing Outlook 57, no. 5: 281–288. 10.1016/j.outlook.2009.07.003. 19789006

[nhs70377-bib-0045] Sharafi, S. , M. A. Cheraghi , A. Nasiri , and G. Mahmoudirad . 2021. “Factors Affecting the Emergence of Diplomacy in Iranian Nurse Managers: A Qualitative Study.” International Nursing Review 68, no. 3: 380–387. 10.1111/inr.12655. 33459367

[nhs70377-bib-0046] Solheim, K. , J. LeClair , B. Pinekenstein , and S. J. Zahner . 2024. “Strategies for Academic Nursing to Advance Global and Planetary Health: A Call to Action.” Journal of Professional Nursing 53: 147–156. 10.1016/j.profnurs.2024.03.005. 38997194

[nhs70377-bib-0013] Stewart, D. , and International Council of Nurses (ICN) . 2022. Nurses: A Voice to Lead Invest in Nursing and Respect Rights to Secure Global Health. ICN. https://www.icn.ch/resources/publications‐and‐reports/nurses‐voice‐lead‐invest‐nursing‐and‐respect‐rights‐secure.

[nhs70377-bib-0047] Stewart, D. , and B. Halpin . 2019. “In Times of Great Need, Great Leaders Emerge ‐ The Shining Voice of Nursing Leaders.” International Nursing Review 66, no. 1: 4–6. 10.1111/inr.12507. 30838651

[nhs70377-bib-0048] Swapna, V. , T. Meera , and K. Nirmala . 2023. “Role of Nurse Leadership to Evaluate the Current Status of Nurses With a View to Invest in Nursing to Secure Global Health – An Exploratory Approach.” Nursing Journal of India CXIV, no. 1: 9–12.

[nhs70377-bib-0049] Tricco, A. C. , E. Lillie , W. Zarin , et al. 2018. “PRISMA Extension for Scoping Reviews (PRISMA‐ScR): Checklist and Explanation.” Annals of Internal Medicine 169, no. 7: 467–473. 10.7326/M18-0850. 30178033

[nhs70377-bib-0050] Wilson, L. , I. A. C. Mendes , H. Klopper , et al. 2016. ““Global Health” and “Global Nursing”: Proposed Definitions From the Global Advisory Panel on the Future of Nursing.” Journal of Advanced Nursing 72, no. 7: 1529–1540. 27062286 10.1111/jan.12973

[nhs70377-bib-0051] World Health Organization . 2014. “Investing in the Power of Nurse Leadership: What Will It Take?” https://www.who.int/publications/m/item/investing‐in‐the‐power‐of‐nurse‐leadership.

[nhs70377-bib-0052] World Health Organization . 2020. State of the World's Nursing. Investing in Education, Jobs and Leadership. World Health Organization, https://www.who.int/publications‐detail/nursing‐report‐2020.

[nhs70377-bib-0053] World Health Organization . 2025. Special Initiative for Action on the Social Determinants of Health for Advancing Health Equity License CC BY‐NC‐SA 3.0 IGO, https://www.who.int/initiatives/action‐on‐the‐social‐determinants‐of‐health‐for‐advancing‐equity.

[nhs70377-bib-0054] World Health Organization (WHO) . 2025. State of the World's Nursing 2025: Investing in Education, Jobs, Leadership and Service Delivery. World Health Organization, https://iris.who.int/server/api/core/bitstreams/a4173924‐a18f‐49b6‐8bd1‐9c2a4a098980/content.

[nhs70377-bib-0055] Zago, P. T. N. , F. C. Mattioni , M. N. Meneses , et al. 2023. “Global Health in Graduate Nursing Programs in Brazil: Contemporary Challenges.” Redin 12, no. 2: 278–292. https://seer.faccat.br/index.php/rediNÃO. ABORDArticle/view/2890.

[nhs70377-bib-0056] Zalon, M. L. , R. Ludwick , and R. M. Patton . 2024. “Strengthening Nurses' Influence in Health Policy.” American Journal of Nursing 124, no. 9: 28–36. 10.1097/01.NAJ.0001028316.80475.bf. 39115377

