# Supplementary material for: A Scoping Review of Nursing Leadership Role in Global Health: Challenges and Opportunities
Source: Nurs Health Sci. 2026 Jul 8;28(3):e70377. doi: 10.1111/nhs.70377 (PMC13346343; doi:10.1111/nhs.70377)
Supplement: Supplementary file 2 — File S2: Search strategy. [file NHS-28-e70377-s001.docx]

**Supplementary file 2: Search strategy**

| **Database** | **Database Provider** | **Search strategy** | **Results** |
| --- | --- | --- | --- |
| PubMed/  MedLine | https://pubmed.ncbi.nlm.nih.gov | ("Global Health"[All Fields] OR "health global"[All Fields] OR "International Health Problems"[All Fields] OR "health problem international"[All Fields] OR "health problems international"[All Fields] OR "International Health Problem"[All Fields] OR "problem international health"[All Fields] OR "problems international health"[All Fields] OR "World Health"[All Fields] OR "health world"[All Fields] OR "Worldwide Health"[All Fields] OR "health worldwide"[All Fields] OR "International Health"[All Fields] OR "health international"[All Fields] OR ("Global Health"[MeSH Terms] OR ("global"[All Fields] AND "health"[All Fields]) OR "Global Health"[All Fields] OR ("healths"[All Fields] AND "international"[All Fields])) OR ("Global Health"[MeSH Terms] OR ("global"[All Fields] AND "health"[All Fields]) OR "Global Health"[All Fields] OR ("international"[All Fields] AND "healths"[All Fields]))) AND ("leadership"[MeSH Terms] OR "leadership"[All Fields] OR "leadership s"[All Fields] OR "leaderships"[All Fields] OR ("leadership"[MeSH Terms] OR "leadership"[All Fields] OR "influential"[All Fields] OR "influentials"[All Fields]) OR "Nursing Leadership"[All Fields]) AND ("Nurse's Role"[All Fields] OR "Nurse's Roles"[All Fields] OR ("Nurse's Role"[MeSH Terms] OR ("nurse s"[All Fields] AND "role"[All Fields]) OR "Nurse's Role"[All Fields] OR ("role"[All Fields] AND "nurse s"[All Fields])) OR ("Nurse's Role"[MeSH Terms] OR ("nurse s"[All Fields] AND "role"[All Fields]) OR "Nurse's Role"[All Fields] OR ("roles"[All Fields] AND "nurse s"[All Fields])) OR "Nurses Role"[All Fields] OR "Nurses Roles"[All Fields] OR "role nurses"[All Fields] OR "roles nurses"[All Fields] OR "Nurse's Scope of Practice"[All Fields] OR ("Nurse's Role"[MeSH Terms] OR ("nurse s"[All Fields] AND "role"[All Fields]) OR "Nurse's Role"[All Fields] OR ("nurse"[All Fields] AND "scope"[All Fields] AND "practice"[All Fields])) OR ("Nurse's Role"[MeSH Terms] OR ("nurse s"[All Fields] AND "role"[All Fields]) OR "Nurse's Role"[All Fields] OR ("nurses"[All Fields] AND "scope"[All Fields] AND "practice"[All Fields])) OR ("Nurse's Role"[MeSH Terms] OR ("nurse s"[All Fields] AND "role"[All Fields]) OR "Nurse's Role"[All Fields] OR ("practice"[All Fields] AND "nurse s"[All Fields] AND "scope"[All Fields])) OR ("Nurse's Role"[MeSH Terms] OR ("nurse s"[All Fields] AND "role"[All Fields]) OR "Nurse's Role"[All Fields] OR ("practice"[All Fields] AND "nurse s"[All Fields] AND "scopes"[All Fields])) OR "Nurses Role"[All Fields] OR "Nurse Role"[All Fields] OR "Nurses Roles"[All Fields] OR "role nurses"[All Fields] OR "roles nurses"[All Fields]) | 197 |
| Cinahl | http://www.ebsco.com | (“Global Health” OR “Health, Global” OR “International Health Problems” OR “Health Problem, International” OR “Health Problems, International” OR “International Health Problem” OR “Problem, International Health” OR “Problems, International Health” OR “World Health” OR “Health, World” OR “Worldwide Health” OR “Health, Worldwide” OR “International Health” OR “Health, International” OR “Healths, International” OR “International Healths”) AND (Leadership OR Influentials OR “Nursing Leadership”) AND (“Nurse's Role” OR “Nurse's Roles” OR “Role, Nurse's” OR “Roles, Nurse's” OR “Nurses Role” OR “Nurses Roles” OR “Role, Nurses” OR “Roles, Nurses” OR “Nurse's Scope of Practice” OR “Nurse Scope, Practice” OR “Nurses Scope, Practice” OR “Practice Nurse's Scope” OR “Practice Nurse's Scopes” OR “Nurses' Role” OR “Nurse Role” OR “Nurses' Roles” OR “Role, Nurses'” OR “Roles, Nurses”) | 52 |
| VHL (LILACS + BDEnf) | https://bvsalud.org | (saúde global OR problemas internacionais de saúde OR saúde internacional OR saúde mundial OR global health OR salud global) AND (liderança OR leadership OR liderazgo OR liderança de enfermagem) AND (papel do profissional de enfermagem OR escopo de prática de enfermagem OR papel da enfermeira OR papel do auxiliar de enfermagem OR papel do enfermeiro OR papel do técnico em enfermagem OR papel dos enfermeiros OR papéis dos enfermeiros OR perfil de competências de enfermeiros OR prática do âmbito do enfermeiro) AND ( db:("BDENF" OR "LILACS")) | 3 |
| Embase | https://www.embase.com/landing | ('global health'/exp OR 'global health' OR 'health, global' OR 'international health problems' OR 'health problem, international' OR 'health problems, international' OR 'international health problem' OR 'problem, international health' OR 'problems, international health' OR 'world health'/exp OR 'world health' OR 'health, world' OR 'worldwide health'/exp OR 'worldwide health' OR 'health, worldwide' OR 'international health'/exp OR 'international health' OR 'health, international' OR 'healths, international' OR 'international healths' OR 'world-wide health'/exp OR 'world-wide health') AND ('leadership'/exp OR leadership OR influentials OR 'nursing leadership' OR 'leader'/exp OR leader) AND ('nurses role'/exp OR 'nurses role' OR 'nurses roles' OR 'role, nurses' OR 'roles, nurses' OR 'nurses scope of practice' OR 'nurse scope, practice' OR 'nurses scope, practice' OR 'practice nurses scope' OR 'practice nurses scopes' OR 'nurse role' OR 'nurses atitude' OR 'nurse atitude') | 185 |
| Scopus | https://www.scopus.com/home.uri | (TITLE-ABS-KEY ( "Global Health" OR "Health, Global" OR "International Health Problems" OR "Health Problem, International" OR "Health Problems, International" OR "International Health Problem" OR "Problem, International Health" OR "Problems, International Health" OR "World Health" OR "Health, World" OR "Worldwide Health" OR "Health, Worldwide" OR "International Health" OR "Health, International" OR "Healths, International" OR "International Healths" ) AND TITLE-ABS-KEY ( leadership OR influentials OR "Nursing Leadership" ) AND TITLE-ABS-KEY ( "Nurse's Role" OR "Nurse's Roles" OR "Role, Nurse's" OR "Roles, Nurse's" OR "Nurses Role" OR "Nurses Roles" OR "Role, Nurses" OR "Roles, Nurses" OR "Nurse's Scope of Practice" OR "Nurse Scope, Practice" OR "Nurses Scope, Practice" OR "Practice Nurse's Scope" OR "Practice Nurse's Scopes" OR "Nurses' Role" OR "Nurse Role" OR "Nurses' Roles" OR "Role, Nurses'" OR "Roles, Nurses" )) | 106 |
| Web of Science | https://access.clarivate.com | ((ALL=((“Global Health” OR “Health, Global” OR “International Health Problems” OR “Health Problem, International” OR “Health Problems, International” OR “International Health Problem” OR “Problem, International Health” OR “Problems, International Health” OR “World Health” OR “Health, World” OR “Worldwide Health” OR “Health, Worldwide” OR “International Health” OR “Health, International” OR “Healths, International” OR “International Healths”) )) AND ALL=((Leadership OR Influentials OR “Nursing Leadership”) )) AND ALL=((“Nurse's Role” OR “Nurse's Roles” OR “Role, Nurse's” OR “Roles, Nurse's” OR “Nurses Role” OR “Nurses Roles” OR “Role, Nurses” OR “Roles, Nurses” OR “Nurse's Scope of Practice” OR “Nurse Scope, Practice” OR “Nurses Scope, Practice” OR “Practice Nurse's Scope” OR “Practice Nurse's Scopes” OR “Nurses' Role” OR “Nurse Role” OR “Nurses' Roles” OR “Role, Nurses'” OR “Roles, Nurses”) ) | 9 |
| Scielo Citation Index (Web of Science) | https://www.scielo.br | TS=((Saúde Global OR Problemas Internacionais de Saúde OR Saúde Internacional OR Saúde Mundial OR Global Health OR Salud Global) AND (Liderança OR Leadership OR Liderazgo OR Liderança de Enfermagem) AND (Papel do Profissional de Enfermagem OR Escopo de Prática de Enfermagem OR Papel da Enfermeira OR Papel do Auxiliar de Enfermagem OR Papel do Enfermeiro OR Papel do Técnico em Enfermagem OR Papel dos Enfermeiros OR Papeis dos Enfermeiros OR Perfil de Competências de Enfermeiros OR Prática do âmbito do Enfermeiro)) | 5 |

# 
