# Supplementary material for: A Scoping Review of Nursing Leadership Role in Global Health: Challenges and Opportunities
Source: Nurs Health Sci. 2026 Jul 8;28(3):e70377. doi: 10.1111/nhs.70377 (PMC13346343; doi:10.1111/nhs.70377)
Supplement: Supplementary file 3 — File S3: Reasons for exclusion. [file NHS-28-e70377-s002.docx]

Supplementary file 3: **Reasons for exclusion**

| **CODE** | **Author (year)** | **1st Reviewer** | **2nd Reviewer** | **3rd Reviewer** | **Inclusion / Exclusion** | **Reasons for exclusion** |
| --- | --- | --- | --- | --- | --- | --- |
| #1 | Opollo (2012) | Include | Include | N/A | **INCLUSION** | N/A |
| #2 | Nicholas (2017) | Include | Include | N/A | **INCLUSION** | N/A |
| #3 | Foster (2018) | Include | Exclude - Out of scope | Exclude - Out of scope | EXCLUSION | Does not address the topic |
| #4 | Salvage (2019) | Exclude - Out of scope | Include | Include | **INCLUSION** | N/A |
| #5 | Schenk (2019) | Include | Include | N/A | **INCLUSION** | N/A |
| #6 | Manson (2021) | Exclude - Out of scope | Exclude - Out of scope | N/A | EXCLUSION | Does not address the topic |
| #7 | Mcmuray (2007) | Include | Include | N/A | **INCLUSION** | N/A |
| #8 | Ferguson (2016) | Exclude - Out of scope | Exclude - Out of scope | N/A | EXCLUSION | Does not address the topic |
| #9 | Baernholdt (2010) | Exclude - Out of scope | Include | Exclude - Out of scope | EXCLUSION | Does not address the topic |
| #10 | Buckner et al. (2014) | Include | Include | N/A | **INCLUSION** | N/A |
| #11 | Rosa (2021) | Exclude - Out of scope | Include | Exclude - Out of scope | EXCLUSION | Does not address the topic |
| #12 | Schneider et al (2009) | Include | Include | N/A | **INCLUSION** | N/A |
| #13 | Swapna et al (2023) | Include | Include | N/A | **INCLUSION** | N/A |
| #14 | Salvage et al (2020) | Include | Include | N/A | **INCLUSION** | N/A |
| #15 | Stewart et al (2019) | Include | Include | N/A | **INCLUSION** | N/A |
| #16 | Bryant-Lukosius et al (2022) | Include | Include | N/A | **INCLUSION** | N/A |
| #17 | Gulzar (2011) | Exclude - Out of scope | Exclude - Out of scope | N/A | EXCLUSION | Does not address the topic |
| #18 | Dawson et al (2015) | Include | Exclude - Out of scope | Include | **INCLUSION** | N/A |
| #19 | Sarna (2005) | Exclude - Out of scope | Exclude - Out of scope | N/A | EXCLUSION | Does not address the topic |
| #20 | Mitrea (2019) | Exclude - Out of scope | Exclude - Out of scope | N/A | EXCLUSION | Does not address the topic |
| #21 | Kim (2006) | Include | Include | N/A | **INCLUSION** | N/A |
| #22 | Salvage (2019b) | Include | Include | N/A | **INCLUSION** | N/A |
| #23 | Mendes (2016) | Exclude - Editorial | Exclude - Editorial | N/A | EXCLUSION | Editorial |
| #24 | Mendes(2016b) | Include | Include | N/A | **INCLUSION** | N/A |
| #25 | Klopper (2019) | Include | Include | N/A | **INCLUSION** | N/A |
| #26 | Potter (2019) | Include | Exclude - Out of scope | Include | **INCLUSION** | N/A |
| #27 | Duncan (2010) | Include | Include | N/A | **INCLUSION** | N/A |
| #28 | Mendes (2020) | Include | Exclude - Out of scope | Include | **INCLUSION** | N/A |
| #29 | Layden (2024) | Exclude - Out of scope | Exclude - Out of scope | N/A | EXCLUSION | Does not address the topic |
| #30 | Porta (2019) | Include | Include | N/A | **INCLUSION** | N/A |
| #31 | Adams (2019) | Exclude - Out of scope | Exclude - Out of scope | N/A | EXCLUSION | Does not address the topic |
| #32 | Adelman (2019) | Exclude - Book Chapter | Exclude - Book Chapter | N/A | EXCLUSION | Book Chapter |
| #33 | Rosa (2021) | Exclude - Out of scope | Exclude - Out of scope | N/A | EXCLUSION | Does not address the topic |
| #34 | Jones (2023) | Include | Exclude - Out of scope | Include | **INCLUSION** | N/A |
| #35 | Rosa (2020) | Exclude - Editorial | Exclude - Editorial | N/A | EXCLUSION | Editorial |
| #36 | Catton (2021) | Exclude - Unavailable in full | Exclude - Unavailable in full | N/A | EXCLUSION | Unavailable in full |
| #37 | Al Ismaili (2023) | Exclude - Out of scope | Exclude - Out of scope | N/A | EXCLUSION | Does not address the topic |
| #38 | Solheim (2024) | Include | Include | N/A | **INCLUSION** | N/A |
| #39 | Backes (2024) | Exclude - Out of scope | Exclude - Out of scope | N/A | EXCLUSION | Does not address the topic |
| #40 | Zalon (2024) | Include | Include | N/A | **INCLUSION** | N/A |
| #41 | Schenk (2021) | Exclude - Out of scope | Exclude - Out of scope | N/A | EXCLUSION | Does not address the topic |
